# Complements and the Wound Healing Cascade: An Updated Review

**DOI:** 10.1155/2013/146764

**Published:** 2013-07-24

**Authors:** Hani Sinno, Satya Prakash

**Affiliations:** Division of Plastic and Reconstructive Surgery, Department of Surgery, McGill University, Montreal, QC, H3A 2B4, Canada

## Abstract

Wound healing is a complex pathway of regulated reactions and cellular infiltrates. The mechanisms at play have been thoroughly studied but there is much still to learn. The health care system in the USA alone spends on average 9 billion dollars annually on treating of wounds. To help reduce patient morbidity and mortality related to abnormal or prolonged skin healing, an updated review and understanding of wound healing is essential. Recent works have helped shape the multistep process in wound healing and introduced various growth factors that can augment this process. The complement cascade has been shown to have a role in inflammation and has only recently been shown to augment wound healing. In this review, we have outlined the biology of wound healing and discussed the use of growth factors and the role of complements in this intricate pathway.

## 1. Introduction

From birth to old age, skin has the vital role of regulating fluid balance, infection control, and thermogenesis. Disruption of this regenerating protective layer can be devastating to the patient and society. More than 2 million burn cases [1] and 7 million chronic skin ulcers caused by pressure, arterial or venous insufficiency, and diabetes mellitus each year in the United States alone are affected by abnormal wound healing [2]. This translates to annual costs of $9 billion in attempt to reduce the major disability and consequent death of such severe skin injury [3]. 

To help reduce patient morbidity and mortality related to abnormal or prolonged skin healing, an understanding of wound healing is essential. Recent works have helped shape the multistep process in wound healing and introduced various growth factors that can augment this process. The complement cascade has been shown to have a role in inflammation and has only recently been shown to augment wound healing (Figure 3). In this work, we will review the biology of wound healing and discuss the use of growth factors and the role of complements in this intricate pathway. 

## 2. Wound Healing

**Figure 1:**
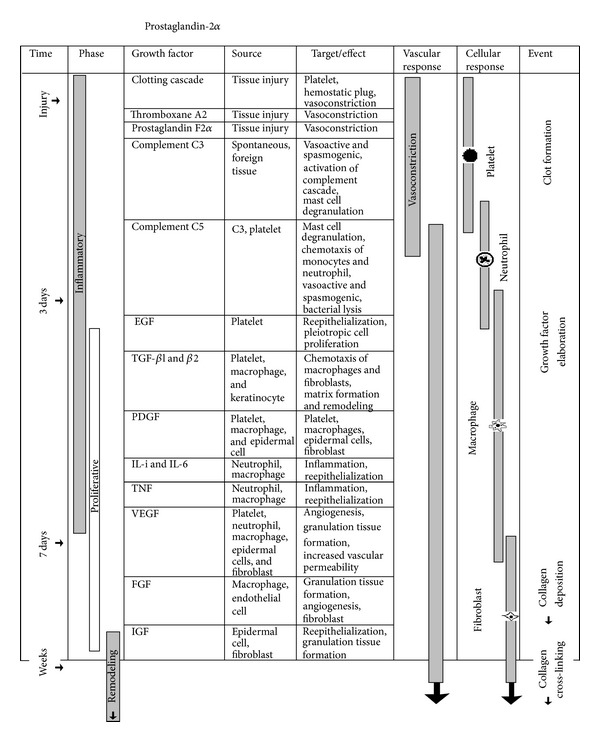
Cytokines and complements involved in inflammation. The three phases of wound healing are associated with different growth factors and subsequent cellular infiltration. Although the complement system is involved in inflammation, its role in wound healing has never been proposed. Complements C3 and C5, epidermal growth factor (EGF), transforming growth factor (TGF), platelet-derived growth factor (PDGF), tumor necrosis factor (TNF), vascular endothelial growth factor (VEGF), and insulin-like growth factor (IGF).

Normal wound healing is a dynamic series of events involving the coordinated interaction of blood cells, proteins, proteases, growth factors, and extracellular matrix components. The wound healing process can be divided into three phases: (1) inflammatory phase; (2) proliferative phase; and (3) maturational phase. Although different predominant cells characterize these phases at differing times, a considerable amount of overlap can occur (Figure 1). 

### 2.1. Inflammatory Phase

The inflammatory phase is the first phase of wound healing and is characterized by hemostasis and inflammation. Hemostasis is initiated during the exposure of collagen during wound formation that activates the intrinsic and extrinsic clotting cascade. In addition, the injury to tissue causes a release of thromboxane A2 and prostaglandin 2-alpha to the wound bed causing a potent vasoconstrictor response. Furthermore, the extravasation of blood constituents provides the formation of the blood clot reinforcing the hemostatic plug. This initial response helps to limit hemorrhage and provides an initial extracellular matrix for cell migration. 

Platelets are among the first response cells that play a key role in the formation of the hemostatic plug. They secrete several chemokines such as epidermal growth factor (EGF), fibronectin, fibrinogen, histamine, platelet-derived growth factor (PDGF), serotonin, and von Willebrand factor. These factors help stabilize the wound through clot formation and also attract and activate macrophages and fibroblasts [4]. They also act to control bleeding and limit the extent of injury. Platelet degranulation activates the complement cascade, specifically C5, a potent neutrophils chemotactic protein [5]. Vasoactive mediators and chemokines are released by the activated coagulation cascade, complement pathways, and parenchymal cells which play a key role in the recruitment of inflammatory leukocytes to injured skin [6].

After hemostasis is achieved, capillary vasodilatation and leakage result secondary to local histamine release by the activated complement cascade. The increased blood flow and altered vascular permeability allow for the migration of inflammatory cells to the wound bed. The presence of foreign organisms further stimulates the activation of the alternate complement pathway. Complement C3 activation results in a cascade of nonenzymatic protein cleavage and interactions that eventually stimulate inflammatory cells and the lysis of bacteria.

The second response cell to migrate to the wound after complement activation and platelet recruitment is the neutrophil. It is responsible for debris scavenging, complement-mediated opsonization and lysis of foreign organisms, and bacterial destruction via oxidative burst mechanisms (i.e., superoxide and hydrogen peroxide formation). Neutrophils kill bacteria and decontaminate the wound from foreign debris. These wastes are later extruded with the eschar or phagocytosed by macrophages.

Macrophages are important phagocytic cells that play a key role in wound healing. They are formed from monocytes stimulated by fragments of the extracellular matrix protein, transforming growth factor *β*, and monocyte chemoattractant protein 1 [7]. In addition to direct phagocytosis of bacteria and foreign materials, macrophages secrete numerous enzymes and cytokines; collagenases, which debride the wound; interleukins and tumor necrosis factor (TNF), which stimulate fibroblasts and promote angiogenesis; and transforming growth factor (TGF), which stimulates keratinocytes [8]. Macrophages also secrete platelet-derived growth factor and vascular endothelial growth factor which initiate the formation of granulation tissue and thus initiate the transition into the proliferative phase and tissue regeneration [6].

### 2.2. Proliferative Phase

The proliferative phase is marked by epithelialization, angiogenesis, granulation tissue formation, and collagen deposition. Epithelialization occurs within hours after injury in wound repair. With an intact basement membrane, the epithelial cells migrate upwards in the normal pattern as occurs in a first-degree skin burn whereby the epithelial progenitor cells remain intact below the wound and the normal layers of epidermis are restored in 2-3 days. If the basement membrane has been damaged, similar to a deeper burn, then the normal epidermal cells from skin appendages (e.g., hair follicles, sweat glands) and the wound periphery reepithelialize the wound.

Neovascularization is necessary to deliver nutrients to the wound and help maintain the granulation tissue bed. Angiogenesis has been attributed to many molecules including fibroblast growth factor, vascular endothelial growth factor, transforming growth factor *β*, angiogenin, angiotropin, angiopoietin 1, tumor necrosis factor alpha, and thrombospondin [9–11]. In different clinical scenarios such as diabetes and vascular disease, this critical nutrient supply by capillaries is insufficient to sustain the tissue deposition in the granulation phase and thus results in a chronically unhealed wound.

The proliferative phase ends with granulation tissue formation. This new stroma begins to invade the wound space close to four days after injury. The new blood vessels at this time have provided a facilitated entry point into the wound to cells such as macrophages and fibroblasts. Macrophages continue to supply growth factors stimulating further angiogenesis and fibroplasia. The secreted platelet-derived growth factor [4] and transforming growth factor *β* [12] along with the extracellular matrix molecules [13] stimulate fibroblasts differentiation to produce ground substance and then collagen. Fibroblasts are the key players in the synthesis, deposition, and remodeling of the extracellular matrix providing strength and substance to the wound.

### 2.3. Maturational Phase

The third and final phase of wound healing is the maturational phase. This is characterized by the transition from granulation tissue to scar formation. Close to two weeks after injury, the wound undergoes contraction, ultimately resulting in a smaller amount of apparent scar tissue. Collagen deposition by fibroblasts continues for a prolonged period with a net increase in collagen deposition reached after three weeks from tissue injury. The entire process is a dynamic continuum dictated by numerous growth factors and cells with an overlap of each of the three phases of wound healing to provide continued remodeling. The human wound is estimated to reach its maximal strength at one year, with a maximal tensile strength that is 70% of normal skin [14].

### 2.4. Healing of Tendons, Ligaments, Bones, and Muscles

Wound healing of tendons, ligaments, bones, and muscles is generally similar to that of skin and other tissues which include the three different phases of inflammation, proliferation, and maturation. Certainly, many of the same growth factors are also involved in these intricate processes. 

#### 2.4.1. Tendons and Ligaments

Heal through “extrinsic” and “intrinsic” processes. The former healing process is based on immobilization whereby fibroblast-dependent ingrowth and neovascularization from the tendon sheath take place. This creates fibrosis and adhesion formation incorporating into the site of tendon injury. The growth factors which stimulate fibroblast are similar to those found in the skin wound healing process described above. Intrinsic healing involved inflammation, proliferation, and remodeling which occur from within the tendon itself. Blood clot and granulation tissue fill the tendon and ligament gaps, Epitenon cells proliferate and transform into fibroblasts, and the wound healing cascade continues to form a stable scar bridging the injured gaps. 

#### 2.4.2. Muscles

Skeletal muscles cells do not regenerate once torn. A similar scar formation can occur to bridge injured gaps.

#### 2.4.3. Bone

Has the unique property of “Creeping Substitution” in which a process in a fracture site or necrotic bone is resorbed and replaced by new tissue moving along channels created by invading host blood vessels found within the periosteum and Volkmann's canals. This process is governed by osteoblasts, osteoclasts and the arcade of cytokines, and growth factors including bone morphogeneic proteins (BMPs), TGF-*β*, fibroblast growth factors (BFGFs), PDGF, and interleukins.

## 3. Complements

**Figure 2:**
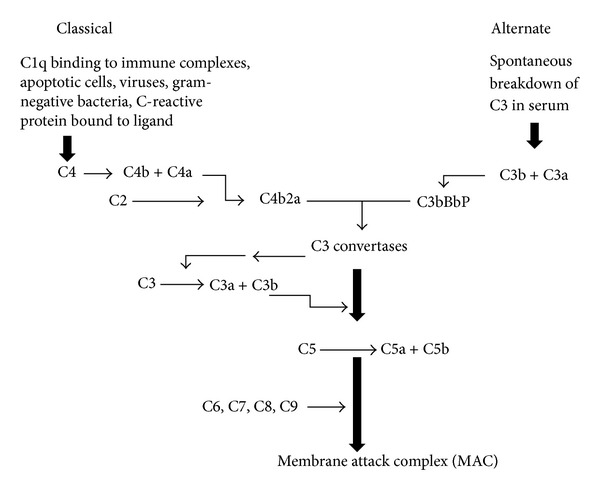
Complement cascade. The complement system converges with the activation of complements C3 and C5 with the subsequent formation of the membrane attack complex. The C3a and C5a proteins are responsible for chemotaxis. The C3b and C5b are responsible for the continued proliferation of the complement cascade.

**Figure 3:**
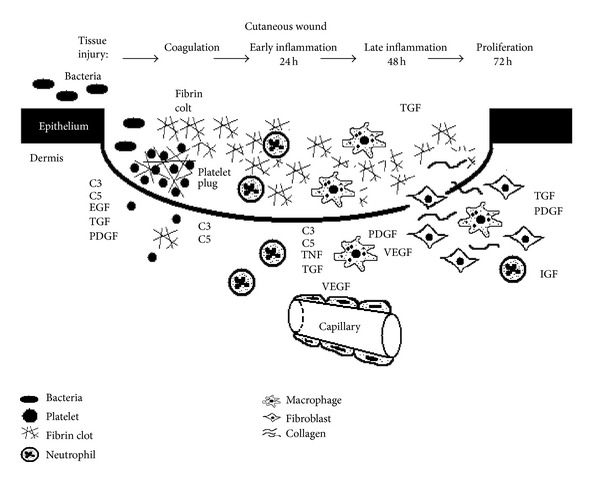
Cutaneous wound healing in time. A schematic representation of cutaneous wound healing and the growth factors and cellular participants in the first 72 hours of injury. The complement cascade appears to be involved in many stages of the wound healing. Platelets, macrophages, fibroblasts, and the formation of the fibrin clot are the major cellular players in early cutaneous, tendon, ligament, muscle, and bone healing.

The complement system is known to have a profound inflammatory response. It is composed of bactericidal and haemolytic proteins. These eleven molecules interact in two different enzymatic cascades: the classical and the alternative pathways (Figure 2). The resulting membrane attack complex (MAC) is directly responsible for the lysis of foreign organisms. Furthermore, the complement cascade plays an important role in many acute inflammatory processes and host defences.

The *classical pathway* is activated by an antibody bound to a foreign particle. The first component is complement 1 (C1), which can also be activated by IgM and IgG immunoglobulins. C4 then C2 are cleaved to activate C3 and C5 [15]. Through the *lectin pathway*, the mannan-binding protein (MBP) can activate the classical pathway independent of antibody by substituting for C1 [16].

The *alternate pathway* functions in an antibody-independent route and thus does not require prior exposure to a particle for activation. It allows for rapid complement activation and amplification on foreign microorganisms and surfaces. Factors B and D are key proteins that promote the activation of C3 and C5. Damaged tissue may contain proteases capable of proteolytic cleavage of C3 and C5 [17]. C3 is cleaved to form C3a and C3b. C5 also becomes C5a and C5b when activated. The “a” component is generally the chemotactic protein, while the “b” component further stimulates the proliferation of the complement cascade. 

The final enzymatic step in the complement pathway allows for cleavage of C5 and the initiation of MAC formation. The membrane attack complex is composed of C5,6,7,8, and 9 [18]. Complements C5 to C9 are noncovalently bound to form MAC which causes pore development in membranes of microorganisms and consequent lysis and death [18].

Other activators of the complement pathway exist. C-reactive protein (CRP) has been shown to bind damaged host tissue and microorganisms, consequently activating C1 [16]. This activation of the classical pathway of complement enhances phagocyte recruitment. Serum protein amyloid P component also binds damaged cells and activates complement [19]. 

The most common complement protein in human serum is C3. C3a, originally identified as an anaphylatoxin, was shown to act as a mediator of inflammatory reactions with effects on polymorphonuclear leukocytes (PMNLs), vascular tone, and leakage. C3a has spasmogenic activities [20] and increases microvascular permeability [5]. Its effects on wound strength and healing have not yet been shown.

C3a and C5a are released through the proteolytic cleavage in the activation pathways. They have a chemotactic response to neutrophils [21]. C3a and C5a bind to specific receptors on mast cells and basophils and trigger degranulation, releasing inflammatory agents into the tissues. This anaphylactic response is controlled by carboxypeptidase N which cleaves the carboxy-terminal arginine residue from both C3a and C5a [22]. This cleavage prevents the activated complements from binding receptors on mast cells. Cleaved C5a, however, retains its chemotactic activity to recruit neutrophils and activate basophils.

C5a has been shown to be chemotactically active for monocytes and PMNL and also promotes the release of free radicals and tissue-digesting enzymes from these cells [23]. C5a promotes neutrophil migration during acute inflammation [24]. The oxidative burst power of neutrophils is further strengthened by C5a binding allowing for augmented bacterial phagocytosis. C5a also has spasmogenic activities [20] and increases microvascular permeability [5].

## 4. Complements and Wound Healing

Both complements C3 and C5 have been recently shown to augment wound healing [25–27]. Sinno et al. demonstrated that the topical application of C3 in a collagen vehicle can successfully increase wound healing. There was a measured increase in maximal breaking strength by 74% [25]. The authors also found a correlation of increased inflammatory cellular recruitment and increased collagen deposition and organization along with this increase in wound strength. Furthermore, Sinno et al. further demonstrated that the topical application of C5 in collagen vehicle also accelerated wound healing [26]. In fact, C5-treated wounds maximal breaking strength increased by 83 percent at day 3 and by 64 percent at day 7 when compared to sham wounds. They also found an increase in the inflammatory cellular recruitment, which they attributed to the likely mechanism of action of complement wound healing effects. They also found an objective increase in fibroblast infiltration and collagen deposition. Moreover, complements C3 and C5 have both independently and in combination been shown to accelerate and increase wound healing [27]. 

## 5. Current Trends and Growth Factors

With an understanding of the phases of wound healing and the associated inflammatory cytokines such as complements and other growth factors, therapeutic modalities can be investigated for the management of problematic wounds. It is vital to understand that inflammation and wound healing are governed by an array of growth factors and cytokines and the inflammatory cells that produce them (Figure 1). Platelet-activating factor (PAF), transforming growth factor-beta (TGF-*β*), and platelet-derived growth factor (PDGF), for example, have been shown to affect inflammation and repair by their chemotactic activity for monocytes, neutrophils, smooth muscle cells, and fibroblasts [28–30]. 

Current pharmacologic cytokines and growth factors have a limited role in clinical practice. In one clinical study led by Crovetti and colleagues, a platelet gel was used on cutaneous chronic ulcers in twenty-four patients [31]. They hypothesized that the effects of their topically applied hemoproduct would provide a supplemental load of PDGF, TGF-*β*, and vascular endothelial growth factor (VEGF). Although a complete response was only observed in less than half of the patients in the treatment group, in each case, granulation tissue formation increased after the first application of the product. This product still remains under investigation. 

Tumor necrosis factor-alpha (TNF-*α*) has been associated with inhibition of normal wound healing when found in high levels. In chronic wounds, the levels of TNF-*α* have been found to be 100-fold higher when compared to levels in acute wounds [32]. Streit and colleagues demonstrated promising results with infliximab (acting as a TNF-*α* inhibitor) on chronic wounds in a small case series [33]. 

Diabetes is one metabolic disease that has been associated with problematic wounds. It is hypothesized that impaired wound healing in diabetic patients is a result of decreased angiogenesis that may be secondary to a diminished production of vascular endothelial growth factor (VEGF). Galiano and colleagues demonstrated the effects of topical VEGF to have a significant accelerated repair in experimental wounds in diabetic mice [34]. This study demonstrated a potential role for VEGF therapy in the treatment of diabetic complications characterized by impaired neovascularization. Furthermore, Zhang and colleagues revealed that exogenous application of VEGF can increase early angiogenesis and tensile strength in the ischemic wound in a rat model [35].

Platelet-activating factor (PAF) has been studied by Porras-Reyes and Mustoe as a chemotactic agent with no cell proliferative properties [36]. They found an increase in maximal breaking strength of wounds treated with PAF at five and seven days after injury compared to controls. Furthermore, with the PAF receptor antagonist introduction, the PAF response was blocked. This revealed that PAF can promote wound healing, but its endogenous supply is not essential for this purpose.

Transforming growth factor-beta (TGF-*β*) has been described as a potent growth factor involved in wound healing. It has been shown to influence the inflammatory response, angiogenesis, reepithelialization, extracellular matrix deposition, and remodeling [12, 37, 38]. Targeting of TGF-*β* has been shown to accelerate wound healing and reduce scarring [38, 39].

Another precursor for the development of problematic wounds is previous radiotherapy exposure. It has been clinically shown to impair wound healing [40]. These effects were attributed to the diminished hematopoiesis with whole body radiation and local tissue mesenchymal injury with surface irradiation. Mustoe and colleagues observed a fifty percent increase in the strength of radio-treated wounds with the topical application of platelet-derived growth factor-BB homodimers (PDGF-BB) [29]. They attributed this to the increase in macrophage recruitment during the early phases of wound healing.

Currently, only few cytokines and growth factors are used clinically. To date, one of the few available commercial products proven to be efficacious in randomized, double-blind-studies is PDGF [41–43]. In multiple studies, recombinant human PDGF-BB has been demonstrated to reduce healing time and improve the incidence of complete wound healing in stages III and IV ulcers. Within the realm of bone healing, BMP have also had great promise. The delivery of these active peptides has also had great research and has been shown to influence the healing rates [44].

The limitations of current wound healing treatments call for an urgent development of a safe, effective, and economical formulation for use in wound healing that is associated with so many other diseases and a variety of the population. An effective therapy would ideally decrease the bacterial load, establish stringent inflammatory control, and concomitantly increase wound tensile strength. Furthermore, a rigorous search for optimal peptide-carrier combination along with timing of growth factor administration is warranted.

## 6. Conclusion

The wound healing cascade is a complex milieu of regulated pathways, secreted growth factors, cytokines, and inflammatory cells. Throughout the three phases of healing, different growth factors and cells have been shown to be essential during each step. Different cytokines have been clinically studied to help augment wound healing. Recently, complements have been shown to augment wound healing. These naturally secreted proteins have innate hemostatic, antibacterial, and inflammatory effects that have now been shown to also accelerate wound healing. We recommend further human phase I clinical trials to further understand the role of complements and other growth factors in wound healing.
